# Treatment and surgical factors associated with longer-term glioblastoma survival: a National Cancer Database study

**DOI:** 10.1093/noajnl/vdaa070

**Published:** 2020-06-04

**Authors:** Sindhoosha Malay, Eashwar Somasundaram, Nirav Patil, Robin Buerki, Andrew Sloan, Jill S Barnholtz-Sloan

**Affiliations:** 1 Department of Population and Quantitative Health Sciences, Case Western Reserve University School of Medicine, Cleveland, Ohio, USA; 2 Research Division, University Hospitals of Cleveland, Cleveland, Ohio, USA; 3 Department of Neurology, University Hospitals of Cleveland, Cleveland, Ohio, USA; 4 Department of Neurological Surgery, University Hospitals of Cleveland, Cleveland, Ohio, USA

**Keywords:** glioblastoma, longer-term survival, subtotal resection, total resection, trimodality therapy

## Abstract

**Background:**

Insufficient data exist to characterize factors associated with longer-term survival of glioblastoma (GBM). A population-based analysis of GBM longer-term survivors (LTS) in the United States was conducted to investigate the association between treatment, demographic, surgical factors, and longer-term survival.

**Methods:**

From the National Cancer Database, GBM patients were identified using ICD-O-3 histology codes 9440-9442/3, 2005–2015 and were divided into routine (≤3 years) and longer-term (>3 years) overall survival (OS) groups. Univariable and multivariable logistic regression analysis was used to assess factors associated with longer-term survival. A subset analysis was performed to further investigate the association of extent of resection and treatment combinations on OS outcomes.

**Results:**

A total of 93 036 patients with GBM met study criteria. Among these patients, 8484 were LTS and 84 552 were routine survivors (RS). When comparing LTS (OS of >3 years) with RS (OS of ≤3 years), younger age, insured status, metro/urban residence, treatment at academic facility, and fewer comorbidities were associated with longer-term survival. In addition, trimodality therapy (chemotherapy + radiation + surgery) was associated with having best odds of longer-term survival (odds ratio = 4.89, 95% confidence interval [3.58, 6.68]); 74% of LTS received such therapy compared with 51% of RS. Subset analysis revealed that total resection is only associated with longer-term survival status for those receiving trimodality therapy or surgery only.

**Conclusions:**

In a population-based analysis, standard of care surgery and chemo radiation connote a survival advantage in GBM. Among those receiving standard of care, having a total resection is most beneficial for longer-term survival status.

**Key Point**

- Standard of care offers a significant survival advantage for glioblastoma patients.

**Importance of the Study**

Glioblastoma is among the most aggressive and lethal primary malignant brain tumors with median survival of 12–14.6 months. In the clinical trial setting, treatment selection and extent of surgical resection (EOR) are considered to be the most important factors that promote better overall survival. The objective of the study is to better understand the strength of association of standard of care (SOC; surgery, radiation, and chemotherapy), EOR, which has been proposed by others to contribute to quality of care to longer-term survival. In our study, we defined longer-term and routine survival groups based on the overall survival. We found patients treated with SOC had a distinct survival advantage compared to those who did not receive SOC. In addition, having total resection is significantly associated with being a longer-term survivor among SOC patients. These results confirm survival benefit for SOC treatment in determining overall survival of glioblastoma patients.

Glioblastoma (GBM) is the most common primary malignant brain tumor in adults representing 48.3% of all malignant brain tumors.^1^ The exact pathogenesis of the condition is poorly understood and could possibly arise from several cell types.^2^ The age-adjusted incidence of GBM is 3.22 per 100 000 with increasing incidence with age and mean age of 65 years at diagnosis^1^ and men have a higher incidence than women.^1^ In terms of race, whites have the highest incidence followed by blacks.^3^

Although new treatment options have emerged since 2005, GBM remains incurable. Without any treatment, the GBM patient can survive maximum up to 3 months. The median survival for patients participating in clinical trials is 14.6 months, while it remains about 12 months overall depending upon the intrinsic tumor factors and treatment combinations and the landmark survival of 16% at 3 years and 10% at 5 years from diagnosis.^4,5^ Younger age of diagnosis and higher Karnofsky Performance Scores (KPS) are strongly associated with longer survival.^6^ Primary tumor location also impacts survival with cerebellar tumors resulting in poorer survival compared to supratentorial tumors.^7^ Extent of resection (EOR) also impacts longer-term survival with those having more than 95% to 98% of the tumor resected having the longest survival.^5,8–11^ In addition, tumor molecular markers, including MGMT promoter methylation and IDH mutation, have been associated with longer survival, with the latter defining a distinct group of “secondary” GBMs.^3,9,11^

Standard of care treatment since 2005 for non-elderly patients consists of maximum safe surgical resection followed by concurrent external beam radiation therapy, typically followed by additional adjuvant chemotherapy,^12,13^ and since 2015, use alternating electrical fields.^14^ This combination treatment with chemotherapy, radiation, and surgery (trimodality therapy) has been shown to improve GBM survival.^5,9–11^

Despite these improvements, modern-day therapy regimens have not drastically improved survival outcomes in the past 40 years.^12^ And yet there are individuals do become longer-term GBM survivors, typically considered 3 years or longer from initial diagnosis. However, factors associated with longer-term survival in GBM patients are not well characterized. In particular, we were interested in whether certain treatment combinations coupled with EOR are associated with a survival advantage, specifically in a broad, inclusive population-based setting.

## Methods

**Figure 1.**
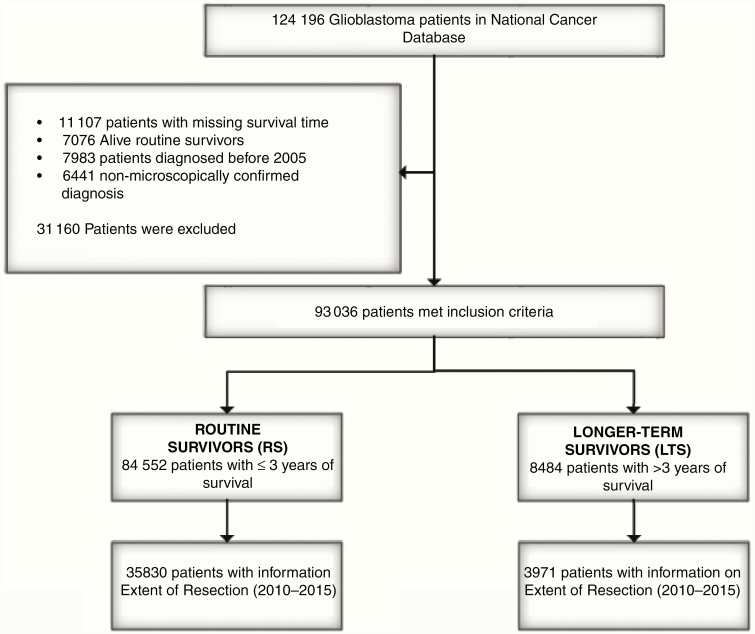
Selection of glioblastoma patients from the National Cancer Database, 2005–2015.

The National Cancer Database (NCDB) collects cancer patient data by registry staff from medical records of Commission on Cancer accredited hospital programs. About 70% of all US cancer diagnoses occur at one of these institutions.^15^ We selected microscopically confirmed patients with ICD-O-3 histology code 9440-9442/3, and diagnostic confirmation 1-4, which represented 124 196 patients in the database. We then removed patients for whom survival data and vital status were missing. Since the current standard of care for GBM consists of maximal, safe surgical resection, concurrent chemo radiation was established in 2005,^15,16^ we also excluded patients diagnosed before 2005. In addition, patients with an “alive” vital status and less than 36 months of survival since diagnosis were removed because they are not evaluable for this study. We only included patients up until 2015 to allow for sufficient follow-up for survival modeling. Our final patient population was 93 036 (Figure 1). We defined longer-term survival to be 36 months and more, which is 4 times the median survival (9.2 months) based on this data set. Patients surviving not more than 36 months were classified as having “routine” survival.

For the descriptive results, race was classified as black, white, or other. Charlson/Deyo score was categorized as 0, 1, 2, and 3+. Facility type was defined to be academic or nonacademic. Facility location was classified as Midwest, Northeast, South, and West. Patient’s residence was defined as rural, urban, or metro. Age of diagnosis was grouped into tranches of younger than 60 years, 60–70 years, and older than 70 years of age. Insurance status was defined as insured or uninsured. Tumor location was classified as infratentorial (C716 and C717), supratentorial (C710–C714), and others (C700, C715, C718, and C719). If a patient received any form of chemotherapy, radiation, or surgery then those treatment categories were classified as “yes.” A treatment modality category was classified as “no” if the database explicitly stated no treatment of that modality was administered. Specific chemotherapy treatments and duration of treatment were not included. To understand in detail about the treatment combinations, we categorized the treatment information as chemotherapy only, radiation only, surgery only, radiation plus chemotherapy, radiation plus surgery, surgery plus chemotherapy, and surgery plus radiation plus chemotherapy. EOR data became available for patients diagnosed 2010 and later who received surgery. EOR was classified as biopsy, subtotal, or total.

Furthermore, a subsequent subset analysis was carried out, and we selected patients who received surgery, were diagnosed from 2010, and had a defined EOR. We performed 2 set of analysis on the subset data, one on overall patients (*n* = 39 801) and other on patients older than 40 years (*n* = 37 771). We excluded patients diagnosed younger than age 40 since their treatment facility information is censored (Supplementary Tables 1 and 2). Patients were stratified by whether they received external beam radiation therapy, chemotherapy, or both modalities (standard of care) following surgery. Additional descriptors of KPS, IDH mutation status, and MGMT promoter methylation status were missing on almost 97% of the GBM patients, as such these variables were not included in the final analysis.

For the descriptive statistics, patient and treatment characteristics of the patients were described using frequency and percentages for categorical variables. Categorical variables were analyzed with the chi-square test. We performed 2 logistic regression analyses using univariable and multivariable models generating odds ratios (ORs) with 95% confidence intervals (95% CI). In the first analysis, we assessed how age, sex, race, ethnicity, Charlson/Deyo score, tumor location, and treatment combination impacted the OR of being a longer-term survivor (LTS). Patients with no treatment were excluded from this analysis, so 86 969 patients were included in the logistic analysis. In the second analysis on patients who received surgery, we wanted to see how EOR is associated with being a longer-term survival, stratified by treatment combinations, adjusting for age, sex, race, ethnicity, Charlson/Deyo score, tumor location, and year of diagnosis. *P* values of less than .05 and OR with CIs excluding 1 were considered statistically significant for the analysis. All the analyses were performed using SAS software, version 9.4 (SAS Institute) and R software, version 3.5.3.

## Results

In total, 93 036 individuals were identified with GBM from NCDB diagnosed between 2005 and 2015 who met study criteria (Figure 1). Among those, 8484 patients were categorized as LTS; the remaining 84 552 patients were categorized as routine survivors (RS). Table 1 presents the baseline characteristics of GBM patients from NCDB diagnosed between 2005 and 2015 according to the survival category (LTS vs RS). We identified significant survival differences in the age, race ethnicity, insurance, patient’s residence, treatment facility, Charlson/Deyo score, tumor location, and treatment combinations. Longer-term survivorship was associated with age (63% younger than 60 years), residence (84% residing in metropolitan areas), comorbidity (83% with a Charlson/Deyo score of 0). In comparison, RS were older (only 35% younger than 60 years), were less likely to reside in metro areas (only 79% residing in metropolitan areas), and had at least one comorbidity (66% with a Charlson/Deyo score of 0). Among LTS, 92% received surgery, 85% received radiation, and 83% received chemotherapy. Among RS, fewer received these therapies: 79% received surgery, 73% received radiation, and 67% received chemotherapy. Table 1 also presents the differences in who received different treatment combinations. The most striking result was “All modalities” (standard of care) where 74% of LTS received it compared with 51% of RS.

**Table 1.** Patient and Treatment Factors of Glioblastoma Patients Based on Longer-Term (>3 years) Versus Routine (≤3 years) Survivors (*n* = 93 036), NCDB 2005–2015

	Overall (*n* = 93 036)	Longer-term survivors (LTS) (*n* = 8484)	Routine survivors (RS) (*n* = 84 552)	*P*
Age at diagnosis (years), *n* (%)				<.001^a^
<60	34 741 (37.3)	5332 (62.8)	29 409 (34.8)	
60–70	30 433 (32.7)	2335 (27.5)	28 098 (33.2)	
70	27 862 (29.9)	817 (9.6)	27 045 (32.0)	
Male, *n* (%)	53 957 (58.0)	4692 (55.3)	49 265 (58.3)	<.001^a^
Race, *n* (%)				<.001^a^
White	84 654 (91.0)	7465 (88.0)	77 189 (91.3)	
Black	5088 (5.5)	556 (6.6)	4532 (5.4)	
Other	3294 (3.5)	463 (5.5)	2831 (3.3)	
Ethnicity, *n* (%)				<.001^a^
Hispanic	4190 (4.5)	564 (6.6)	3626 (4.3)	
Non-Hispanic	83 548 (89.8)	7489 (88.3)	76 059 (90.0)	
Missing	5298 (5.7)	431 (5.1)	4867 (5.8)	
Insurance status, *n* (%)				<.001^a^
Yes	87 735 (94.3)	7832 (92.3)	79 903 (94.5)	
No	3096 (3.3)	409 (4.8)	2687 (3.2)	
Missing	2205 (2.4)	243 (2.9)	1962 (2.3)	
Residence, *n* (%)				<.001^a^
Metro	74 297 (79.9)	7061 (83.2)	67 236 (79.5)	
Urban	14 381 (15.5)	1080 (12.7)	13 301 (15.7)	
Rural	1805 (1.9)	103 (1.2)	1702 (2.0)	
Missing	2553 (2.7)	240 (2.8)	2313 (2.7)	
Treatment location, *n* (%)				<.001^a^
Midwest	22 208 (23.9)	1608 (19.0)	20 600 (24.4)	
Northeast	18 582 (20.0)	1828 (21.5)	16 754 (19.8)	
South	32 856 (35.3)	2668 (31.4)	30 188 (35.7)	
West	14 818 (15.9)	1118 (13.2)	13 700 (16.2)	
Missing	4572 (4.9)	1262 (14.9)	3310 (3.9)	
Treatment facility, *n* (%)				<.001^a^
Academic	38 955 (41.9)	3809 (44.9)	35 146 (41.6)	
Nonacademic	49 509 (53.2)	3413 (40.2)	46 096 (54.5)	
Missing	4572 (4.9)	1262 (14.9)	3310 (3.9)	
Tumor location, *n* (%)				<.001^a^
Infratentorial	977 (1.1)	77 (0.9)	900 (1.1)	
Supratentorial	70 363 (75.6)	6877 (81.1)	63 486 (75.1)	
Others	21 696 (23.3)	1530 (18.0)	20 166 (23.9)	
Charlson/Deyo score, *n* (%)				<.001^a^
0	65 694 (70.6)	6857 (80.8)	58 837 (69.6)	
1	16 303 (17.5)	1064 (12.5)	15 239 (18.0)	
2	7327 (7.9)	405 (4.8)	6922 (8.2)	
≥3	3712 (4.0)	158 (1.9)	3554 (4.2)	
Chemotherapy, *n* (%)				<.001^a^
Yes	62 532 (67.2)	7022 (82.8)	55 510 (65.7)	
No	27 505 (29.6)	1145 (13.5)	26 360 (31.2)	
Missing	2999 (3.2)	317 (3.7)	2682 (3.2)	
Radiation, *n* (%)				<.001^a^
Yes	67 414 (72.5)	7189 (84.7)	60 225 (71.2)	
No	25 157 (27.0)	1205 (14.2)	23 952 (28.3)	
Missing	465 (0.5)	90 (1.1)	375 (0.4)	
Surgery, *n* (%)				<.001^a^
Yes	73 319 (78.8)	7827 (92.3)	65 492 (77.5)	
No	19 658 (21.1)	655 (7.7)	19 003 (22.5)	
Missing	59 (0.1)	2 (0.0)	57 (0.1)	
Treatment, *n* (%)				<.001^a^
None	6067 (6.5)	84 (1.0)	5983 (7.1)	
Chemotherapy only	543 (0.6)	19 (0.2)	524 (0.6)	
Radiation only	2181 (2.3)	41 (0.5)	2140 (2.5)	
Surgery only	13 864 (14.9)	694 (8.2)	13 170 (15.6)	
Radiation and chemotherapy	10 250 (11.0)	485 (5.7)	9765 (11.5)	
Surgery and chemotherapy	2552 (2.7)	223 (2.6)	2329 (2.8)	
Surgery and radiation	5260 (5.7)	310 (3.7)	4950 (5.9)	
All modalities	49 129 (52.8)	6283 (74.1)	42 846 (50.7)	
Missing	3190 (3.4)	345 (4.1)	2845 (3.4)	

Information on treatment facility and treatment location is available only for patients 40 years and older at diagnosis.

All modalities: surgery + radiation + chemotherapy.

Insurance: Yes (Medicaid, Medicare, Other Government, Private Insurance/Managed Care).

Treatment location: Northeast (New England, Middle Atlantic); Midwest (East North Central, West North Central); South (South Atlantic, East South Central, West South Central); West (Mountain, Pacific).

Treatment facility: nonacademic (Community Cancer Program, Comprehensive Community Cancer Program, Integrated Network Cancer Program) and academic (Academic/Research Program).

Tumor location: infratentorial (cerebellum and brain stem), supratentorial (cerebrum and lobes), others (cerebral meninges, ventricles, brain NOS, and overlapping lesion of brain).

^a^Chi-square test.

The following patient and treatment characteristics were found to be associated with better survival for GBM on both univariate and multivariate analyses: younger age, female sex, non-white race, Hispanic, lower Charlson/Deyo score, supratentorial tumors, and bimodality/trimodality therapy (Table 2). Higher odds of being a LTS were seen among patients who received trimodality therapy (OR, 4.89; *P* = <.001), with the next highest survival odds being any kind of bimodality therapy (surgery plus chemotherapy: OR, 3.73; *P* = <.001 and surgery plus radiation: OR, 2.57; *P* = <.001) when compared to radiation-only patients.

**Table 2.** Multivariable Logistic Regression Results of Patient and Treatment Factors Associated With Odds of Longer-Term Survival (>3 years) After Diagnosis With Glioblastoma, NCDB 2005–2015

	Univariable OR (95% CI)	*P*	Multivariable OR (95% CI)	*P*
Age (years)				
70	Reference		Reference	
<60	5.58 (5.17–6.02)	<.0001	4.46 (4.11–4.84)	<.0001
60–70	2.60 (2.39–2.82)	<.0001	2.22 (2.04–2.43)	<.0001
Sex				
Male	Reference		Reference	
Female	1.15 (1.10–1.2)	<.0001	1.19 (1.19–1.31)	<.0001
Race				
White	Reference		Reference	
Black	1.26 (1.15–1.38)	<.0001	1.19 (1.08–1.32)	.0008
Others	1.69 (1.53–1.87)	<.0001	1.58 (1.40–1.79)	<.0001
Ethnicity				
Non-Hispanic	Reference		Reference	
Hispanic	1.56 (1.43–1.72)	<.0001	1.45 (1.31–1.60)	<.0001
Charlson/Deyo score				
≥3	Reference		Reference	
0	2.49 (2.11–2.93)	<.0001	1.92 (1.61–2.28)	<.0001
1	1.53 (1.29–1.82)	<.0001	1.42 (1.18–1.71)	.0001
2	1.28 (1.06–1.55)	.009	1.17 (0.96–1.44)	.1162
Tumor location				
Infratentorial	Reference		Reference	
Supratentorial	1.28 (1.01–1.62)	.0438	1.58 (1.21–2.06)	.0007
Others	0.92 (0.72–1.17)	.5142	1.22 (0.93–1.59)	.1513
Treatment type				
Radiation only	Reference		Reference	
Chemotherapy only	1.89 (1.09–3.29)	.024	1.40 (0.79–2.47)	.2506
Radiation + chemotherapy	2.59 (1.88–3.58)	<.0001	1.88 (1.36–2.61)	.0001
Surgery only	2.75 (2.00–3.78)	<.0001	2.30 (1.66–3.17)	<.0001
Surgery + radiation	3.27 (2.35–4.54)	<.0001	2.57 (1.84–3.58)	<.0001
Surgery + chemotherapy	5.00 (3.56–7.01)	<.0001	3.73 (2.65–5.25)	<.0001
All modalities	7.65 (5.61–10.43)	<.0001	4.89 (3.58 - 6.68)	<.0001

OR, odds ratio; 95% CI, 95% confidence interval.

All modalities: surgery + radiotherapy + chemotherapy.

### Association of EOR and Treatment Status for GBM LTS

To include EOR information in our model, we performed a subset analysis including only those patients who had surgery and were diagnosed between 2010 and 2015 (Table 3). Table 3 presents all patients who had diagnostic information and some form of surgery during this period. Supplementary Table 1 presents the same results except we excluded patients younger than 40 years since NCDB masks treatment facility information for this population. For all treatment combinations, LTS were significantly more likely to have had total resection, whereas RS were significantly more likely to have subtotal resections. Patients treated with the combination of surgery and chemotherapy had nonsignificant differences, likely due to small sample size. In order to further investigate the relationship between EOR, treatment combinations, and survival status, we included only those patients who explicitly had “biopsy,” “subtotal,” or “total” resection listed in their record. From Table 4 (and Supplementary Table 2), the odds of being a LTS were significantly increased for those who received trimodality therapy and had total resection, but not for those who received trimodality therapy and had subtotal resection, as compared to biopsy. Similar results were seen for those receiving surgery only. For those receiving any bimodality treatment combination, having a total or subtotal resection did not have an effect on longer-term survival status compared to biopsy. 

**Table 3.** Descriptive Statistics of Extent of Resection by Treatment Combinations Among Glioblastoma Patients Who Received Surgery, NCDB 2010–2015 (*n* = 39 801)

	All modalities (*n* = 28 587)		*P*	Surgery + radiation (*n* = 2494)		*P*	Surgery + chemo (*n* = 1184)		*P*	Surgery only (*n* = 7536)		*P*
	Longer-term survivors (*n* = 3385)	Routine survivors (*n* = 25 202)		Longer-term survivors (*n* = 116)	Routine survivors (*n* = 2378)		Longer-term survivors (*n* = 96)	Routine survivors (*n* = 1088)		Longer-term survivors (*n* = 374)	Routine survivors (*n* = 7162)	
Extent of resection, *n* (%)			<.001^a^			.008^a^			.624^a^			<.001^a^
Biopsy	580 (17.13)	5024 (19.93)		21 (18.10)	592 (24.89)		20 (20.83)	242 (22.24)		90 (24.06)	1917 (26.77)	
Subtotal	855 (25.26)	7982 (31.67)		28 (24.14)	759 (31.92)		25 (26.04)	323 (29.69)		70 (18.72)	2179 (30.42)	
Total	1950 (57.61)	12 196 (48.39)		67 (57.76)	1027 (43.19)		51 (53.13)	523 (48.07)		214 (57.22)	3066 (42.81)	

All modalities: surgery + radiation + chemotherapy.

^a^Chi-square test.

**Table 4.** Multivariable Logistic Regression Results on Extent of Resection on Glioblastoma Patients Who Received Surgery, Stratified by Treatment Combinations, NCDB 2010–2015

	All modalities		Surgery + radiation		Surgery + chemotherapy		Surgery only	
	Multivariable* OR (95% CI)	*P*	Multivariable* OR (95% CI)	*P*	Multivariable* OR (95% CI)	*P*	Multivariable* OR (95% CI)	*P*
Extent of resection								
Biopsy	Reference		Reference		Reference		Reference	
Subtotal	0.92 (0.82–1.03)	.1658	1.08 (0.59–1.99)	.7996	0.90 (0.47–1.71)	.7419	0.66 (0.46–0.90)	.0097
Total	1.35 (1.22–1.49)	<.001	1.61 (0.93–2.76)	.0844	1.21 (0.68–2.13)	.5157	1.36 (1.04–1.77)	.0163
*Adjusted for age, sex, race, ethnicity, Charlson/Deyo score, and tumor location.								
Extent of resection								
Biopsy	Reference		Reference		Reference		Reference	
Subtotal	1.01 (0.89–1.14)	.8962	1.13 (0.58–2.20)	.7159	1.02 (0.50–2.09)	.9640	0.65 (0.45–0.93)	.0206
Total	1.41 (1.26–1.57)	<.001	1.79 (1.00–3.18)	.0509	1.41 (0.75–2.66)	.2867	1.34 (1.01–1.77)	.0439
*Adjusted for age, sex, race, ethnicity, Charlson/Deyo score, tumor location, and year of diagnosis.								

OR, odds ratio; 95% CI, 95% confidence interval.

All modalities: surgery + radiation + chemotherapy.

## Discussion

The prognosis for GBM is improved for younger, female, and non-white patients. LTS are more likely to be from metropolitan areas and have had treatment at academic treatment facilities. A similar NCDB study on assessing GBM survival on a cohort of patients diagnosed from 2004 to 2009 found similar factors predicting survival.^17^ As expected, a higher Charlson/Deyo score is associated with poorer survival. Increased comorbidity of disease may limit aggressive treatment options and worsen the clinical progression of GBM. From the previous literature, it is established that younger age and fewer comorbidities are associated with better survival,^7,17^ although age at diagnosis may also be a proxy for IDH-mutant secondary GBM. Less explored in literature are how different treatment combinations may affect longer-term survival.

Our data demonstrate that the patients who received the bimodality/trimodality therapy are the most likely to become a LTS. This is consistent with the literature which in general finds that more aggressive therapy is consistent with longer survival.^9–11,18^ Specifically, the LTS were more likely to receive maximal surgical resection, chemotherapy, and radiation, which mirrors the current standard of care for GBM prior to the advent of alternating electrical field therapy.^13,14^

Some patients receive only 1 or 2 modalities of treatment. Among patients who received treatment, those who received radiation only were the least likely to be a LTS followed by those who getting chemotherapy only. Interestingly, surgery-only therapy resulted in better odds of longer-term survival than bimodality treatment with chemotherapy and radiation indicating that surgery is particularly important among the 3 modalities. The treatment combinations with the highest odds of longer-term survival all featured surgery. Surgery plus radiation and surgery plus chemotherapy were both significantly associated with better odds compared with radiation plus chemotherapy. As expected, those who received trimodality treatment had the best odds of longer-term survival.

Given that our analysis of treatment combinations showed a particularly important role of surgery in improving survival odds, we wanted to further explore how EOR was associated with different treatment combinations in affecting survival outcomes in patients who received surgery. The NCDB provided 3 levels of resection: biopsy only, subtotal, and total resection. Subtotal resection was no better than biopsy in patients who received trimodality or bimodality care. Surprisingly, among surgery-only patients, we found that subtotal resection resulted in poorer odds of survival compared to biopsy. This could be due to several reasons. A retrospective review that analyzed tumor volume post-surgery finds that residual tumor volume may be an important prognostic indicator^19^ and may also depend on the extent of subtotal resection threshold.^20^ A similar study in the NCDB found no advantage to subtotal resection over biopsy for overall survival in GBM.^21^ A SEER study in pediatric GBM patients had also found no difference between subtotal EOR and biopsy only.^22^

In all treatment combinations featuring surgery, longer-term survival odds improved with total resection, though this was only significant in surgery-only patients and trimodality therapy (standard of care). Nonsignificance is likely due to sample size effect as most patients receive standard of care compared to bimodality or unimodality treatments.

**Figure 2.**
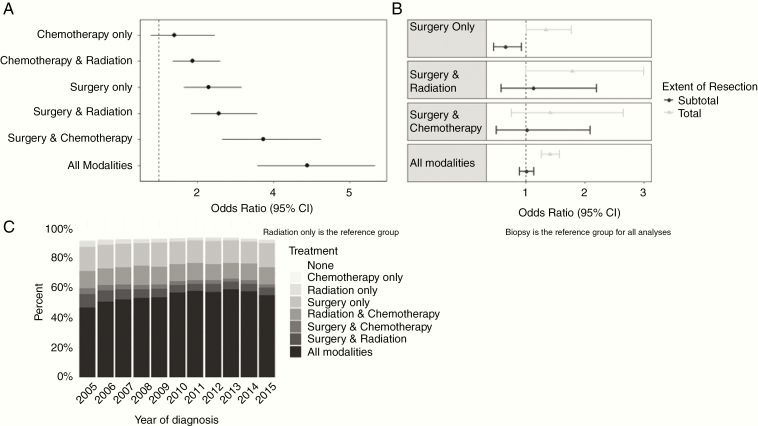
Odds of longer-term survival status with 95% confidence intervals (95% CIs) for patients with glioblastoma by treatment status NCDB 2005–2015 (A) and by extent of resection stratified by treatment status NCDB 2010–2015 (B) and descriptive percentages of treatment combinations by year of diagnosis (C).

We were also interested in whether year of diagnosis impacted longer-term survivorship. We observed that the proportion of patients receiving trimodality (standard of care) treatment generally increased with each year (Figure 2C). However, adjusting our model for year of diagnosis did not significantly change the OR despite a change in treatment patterns from 2005 to 2015. These results are represented in Table 4 and Supplementary Table 2.

Our study utilizes the NCDB’s most up to date data regarding the survival of GBM patients, but does have several limitations. We excluded 2016 patient data as follow-up time was not sufficient for our 3-year criteria for longer-term survival. We observed 1% of the LTS received no treatment at all. A possible explanation for this perplexing finding may be a subset of GBM patients whose disease progresses very slowly and requires no treatment to reach longer-term survival status. Alternatively, with respect to these patients, there may also have been data misentry or misclassification regarding diagnosis. In addition, by excluding patients who received no treatment, we limit the potential for “immortal time” bias where a patient must survive long enough to receive treatment. NCDB masks treatment facility information of patients diagnosed younger than 40, hence we could not adjust for this factor in all models; however, when limiting analyses to patients 40 and older, results were similar (Supplementary Table 2). Since Commission on Cancer accredited facilities are located heavily in metropolitan and urban areas, we did not include patient residence in our logistic model as residence was heavily biased against rural areas (only ~2% of patients were considered rural).^23^ A future study with enough sample size should be carried out to know the associations of these 2 factors on better survival. In addition, we had to exclude certain factors known to strongly affect prognosis due to high levels of missing data in the NCDB, such as KPS and MGMT methylation (information missing for almost 97% of the patients). IDH status is not reported by the NCDB; however, our subset analysis in Supplementary Tables 1 and 2 excludes patients aged 40 and younger, which should limit IDH-mutant cases due to association between young age and IDH-mutant GBM. Including such data may incorporate confounding biases as patients with these markers known are more likely to have been treated at an academic medical center. Another NCDB limitation is mortality, that is “all cause” mortality rather than disease-specific. While this may potentially introduce some bias, it is known that the vast majority of GBM patients die from GBM or related complications, and we do not expect a significant bias from this variable. Regardless, the NCDB is a large comprehensive database providing significant statistical power to tease out specific factors associated with GBM survival. To our knowledge, this is the largest study among GBM patients that examines how treatment combinations and EOR affect longer-term survivorship.

## Conclusions

GBM is an aggressive type of cancer and the most common type of malignant brain tumor. In our study, we identified several patient and treatment factors that improve the odds of being a LTS. The important conclusion from this study is that patients who received standard of care (trimodality therapy) have a survival advantage when compared to the patients who did not receive standard of care. Among standard of care patients, patients having a total resection were more likely to be LTS compared to those with subtotal resections or biopsy.

## Supplementary Material

vdaa070_suppl_Supplementary_Files
