# Supplementary material for: Treatment and surgical factors associated with longer-term glioblastoma survival: a National Cancer Database study
Source: Neurooncol Adv. 2020 Jun 4;2(Suppl 1):1–10. doi: 10.1093/noajnl/vdaa070 (PMC7332237; doi:10.1093/noajnl/vdaa070)
Supplement: vdaa070_suppl_Supplementary_Files [file vdaa070_suppl_supplementary_files.docx]

**SUPPLEMENTAL FILES**

Table 1: Extent of resection by treatment combinations among glioblastoma patients who received surgery, 40 years and older, NCDB 2010-2015. (n = 37,771)

|  | **All modalities (n = 27,015)** | | **p-value** | **Surgery +Radiation (n = 2,404)** | | **p-value** | **Surgery +Chemo (n = 1,112)** | | **p-value** | **Surgery Only (n = 7,240)** | | **p-value** |  |
| --- | --- | --- | --- | --- | --- | --- | --- | --- | --- | --- | --- | --- | --- |
|  | **LTS**  **(n = 2,907)** | **RS**  **(n = 24,108)** |  | **LTS**  **(n = 103)** | **RS**  **(n = 2,301)** |  | **LTS**  **(n = 81)** | **RS**  **(n = 1,031)** |  | **LTS**  **(n = 326)** | **RS**  **(n = 6,914)** |  |  |
| EOR, n (%)  Biopsy  Subtotal  Total | 501 (17.2)  745 (25.6)  1661 (57.1) | 4801 (19.9)  7606 (31.5)  11701 (48.5) | <0.001 ^a^ | 18 (17.5)  24 (23.3)  61 (59.2) | 577 (25.1)  732 (31.8)  992 (43.1) | 0.005 ^a^ | 15 (18.5)  21 (25.9)  45 (55.6) | 233 (22.6)  306 (29.7)  492 (47.7) | 0.392^a^ | 80 (24.5)  58 (17.8)  188 (57.7) | 1847 (26.7)  2104 (30.4)  2963 (42.9) | <0.001 ^a^ |  |
| Abbreviations: a: Chi-square test; EOR: Extent of resection.; LTS: Longer-term survivors; RS: Routine Survivors  All modalities: Surgery + Radiation + Chemotherapy | | | | | | | | | | | | | |

|  | **All modalities** | | **Surgery + Radiation** | | **Surgery + Chemotherapy** | | | **Surgery only** | |
| --- | --- | --- | --- | --- | --- | --- | --- | --- | --- |
|  | **Multivariable* OR (95% CI)** | **p-value** | **Multivariable* OR (95% CI)** | **p-value** | **Multivariable* OR (95% CI)** | **p-value** | **Multivariable* OR (95% CI)** | | **p-value** |
| Extent of Resection  Biopsy  Subtotal  Total | Reference  0.95 (0.84 - 1.07)  1.36 (1.22 - 1.51) | 0.3904  <.001 | Reference  1.00 (0.52 - 1.92)  1.59 (0.90-2.82) | 0.9985  0.1100 | Reference  0.98 (0.48 – 2.00)  1.40 (0.75 - 2.62) | 0.9642  0.2905 | Reference  0.63 (0.44 - 0.91)  1.34 (1.01 - 1.78) | | 0.0127  0.0417 |
| * Adjusted for Age, Sex, Race, Ethnicity, Charlson Deyo Score Tumor Location and Facility Type. | | | | | | | | | |
| Extent of Resection  Biopsy  Subtotal  Total | Reference  1.03 (0.91 – 1.16)  1.44 (1.29 – 1.61) | 0.6815  <.001 | Reference  1.01 (0.56 – 2.15)  1.75 (0.98 – 3.13) | 0.7774  0.0594 | Reference  1.03 (0.50 – 2.12)  1.45 (0.77 – 2.75) | 0.9428  0.2521 | Reference  0.67 (0.46 – 0.96)  1.34 (1.01 – 1.79) | | 0.0276  0.0440 |
| * Adjusted for Age, Sex, Race, Ethnicity, Charlson Deyo Score, Tumor Location, Facility type and Year of diagnosis. | | | | | | | | | |
| Abbreviations: OR, odds ratio; 95% CI, 95% confidence Intervals; All modalities, Surgery + Radiation + Chemotherapy. | | | | | | | | | |

Table 2: Odds of Longer-Term survival associated with extent of resection on GB patients 40 years and older who received surgery, stratified by treatment combinations, NCDB 2010-2015.
